# [Aqua­bis­(nitrato-κ*O*)copper(II)]-μ-{bis­[5-methyl-3-(pyridin-2-yl)-1*H*-pyrazol-4-yl]selenide}-[diaqua­(nitrato-κ*O*)copper(II)] nitrate monohydrate

**DOI:** 10.1107/S1600536812045217

**Published:** 2012-11-14

**Authors:** Maksym Seredyuk, Vadim A. Pavlenko, Elzbieta Gumienna-Kontecka, Turganbay S. Iskenderov

**Affiliations:** a Department of Chemistry, National Taras Shevchenko University, Volodymyrska Street 64, 01601 Kyiv, Ukraine; b Institut de Ciencia Molecular (ICMol), Departament de Quimica Inorganica, Universitat de Valencia, C/ Catedratico Jose Beltran Martinez, 2, 46980 Paterna (Valencia), Spain; c Faculty of Chemistry, University of Wroclaw, 14, F. Joliot–Curie Street, 50383 Wroclaw, Poland

## Abstract

In the title binuclear complex, [Cu_2_(NO_3_)_3_(C_18_H_16_N_6_Se)(H_2_O)_3_]NO_3_·H_2_O, the Cu^II^ ions are penta­coordinated in a tetra­gonal–pyramidal geometry. In both cases, the equatorial planes are formed by a chelating pyrazole-pyridine group, a water mol­ecule and a nitrate O atom, whereas the apical positions are occupied by a water mol­ecule for one Cu^II^ ion and a nitrate O atom for the other. The organic selenide ligand adopts a *trans* configuration with respect to the C–Se–C plane. Numerous inter­molecular O—H⋯O and N—H⋯O hydrogen bonds between the coordinating and lattice water mol­ecules, nitrate anions and pyrazole groups are observed. π–π stacking inter­actions between the pyridine rings [averaged centroid–centroid distance = 3.652 (5) Å] are also present. The lattice water molecule is equally disordered over two sets of sites.

## Related literature

For details and applications of related pyrazole compounds, see: Fritsky *et al.* (2003 ▶); Kovbasyuk *et al.* (2004 ▶); Krämer *et al.* (2002 ▶); Krämer & Fritsky (2000 ▶); Penkova *et al.* (2009 ▶); Sachse *et al.* (2008 ▶). For structural studies of related pyrazolylselenides, see: Seredyuk *et al.* (2010*a* ▶, 2011 ▶, 2012 ▶). For structural studies of *d*-metal complexes with bis­(3,5-dimethyl-1*H*-pyrazol-4-yl)selenide, see: Seredyuk *et al.* (2007 ▶, 2009 ▶, 2010*b* ▶,*c* ▶). For related structures, see: Fritsky *et al.* (2004 ▶); Kanderal *et al.* (2005 ▶); Moroz *et al.* (2010 ▶, 2012 ▶). For the treatment of disordered water mol­ecules, see: Nardelli (1999 ▶).
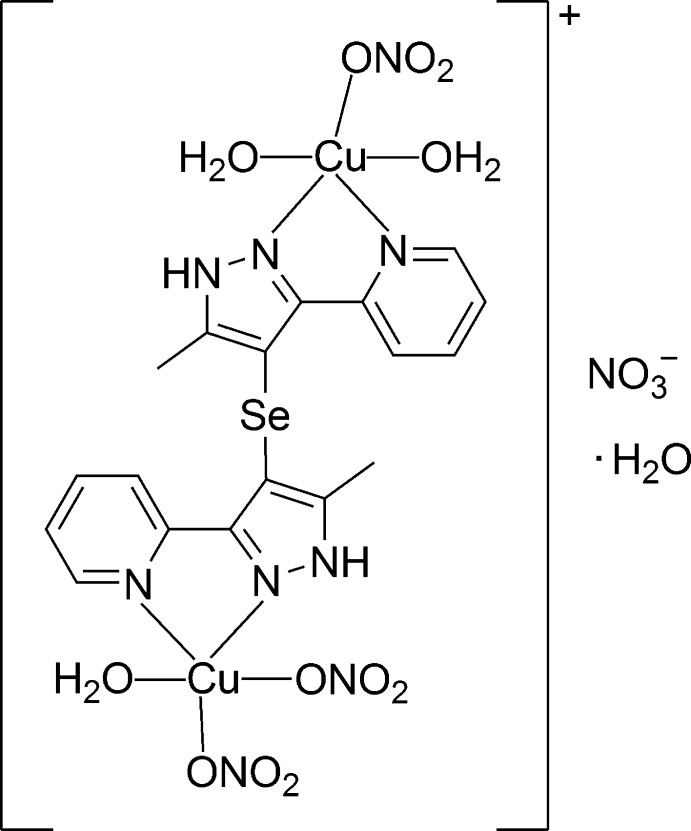


## Experimental

### 

#### Crystal data

[Cu_2_(NO_3_)_3_(C_18_H_16_N_6_Se)(H_2_O)_3_]NO_3_·H_2_O
*M* *_r_* = 842.51
Triclinic, 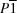

*a* = 10.102 (2) Å
*b* = 11.629 (2) Å
*c* = 12.796 (3) Å
α = 98.56 (3)°
β = 93.07 (3)°
γ = 93.04 (3)°
*V* = 1481.5 (5) Å^3^
*Z* = 2
Mo *K*α radiation
μ = 2.76 mm^−1^
*T* = 100 K
0.27 × 0.23 × 0.13 mm

#### Data collection

Bruker APEXII CCD diffractometer
Absorption correction: multi-scan (*SADABS*; Sheldrick, 1996 ▶) *T*_min_ = 0.476, *T*_max_ = 0.716
11167 measured reflections
6511 independent reflections
5817 reflections with *I* > 2σ(*I*)
*R*_int_ = 0.073

#### Refinement

*R*[*F* ^2^ > 2σ(*F* ^2^)] = 0.080
*wR*(*F* ^2^) = 0.223
*S* = 1.02
6511 reflections
418 parameters
39 restraints
H-atom parameters constrained
Δρ_max_ = 2.05 e Å^−3^
Δρ_min_ = −1.35 e Å^−3^

### 

Data collection: *APEX2* (Bruker, 2007 ▶); cell refinement: *SAINT* (Bruker, 2007 ▶); data reduction: *SAINT*; program(s) used to solve structure: *SIR2004* (Burla *et al.*, 2005 ▶); program(s) used to refine structure: *SHELXL97* (Sheldrick, 2008 ▶); molecular graphics: *DIAMOND* (Brandenburg, 1999 ▶); software used to prepare material for publication: *SHELXL97*.

## Supplementary Material

Crystal structure: contains datablock(s) I, global. DOI: 10.1107/S1600536812045217/hy2595sup1.cif

Structure factors: contains datablock(s) I. DOI: 10.1107/S1600536812045217/hy2595Isup2.hkl

Supplementary material file. DOI: 10.1107/S1600536812045217/hy2595Isup3.cdx

Supplementary material file. DOI: 10.1107/S1600536812045217/hy2595Isup4.cdx

Additional supplementary materials:  crystallographic information; 3D view; checkCIF report

## Figures and Tables

**Table 1:** Hydrogen-bond geometry (Å, °)

*D*—H⋯*A*	*D*—H	H⋯*A*	*D*⋯*A*	*D*—H⋯*A*
O1—H1*O*1⋯O4	0.85	1.86	2.710 (12)	176
O1—H2*O*1⋯O15^i^	1.00	1.77	2.763 (9)	174
O8—H1*O*8⋯O14	0.85	2.02	2.744 (9)	142
O8—H2*O*8⋯O2^ii^	0.94	1.88	2.772 (9)	156
O9—H1*O*9⋯O13	0.85	1.88	2.732 (9)	173
O9—H2*O*9⋯O9^iii^	0.85	1.94	2.794 (9)	175
O9—H3*O*9⋯O1*WA* ^iii^	0.85	2.40	3.024 (18)	131
O9—H3*O*9⋯O1*WB* ^iii^	0.85	1.91	2.723 (16)	159
N2—H1*N*2⋯O14^iv^	0.80	2.08	2.822 (10)	155
N5—H1*N*⋯O1*WA*	0.86	2.04	2.878 (18)	166
O1*WA*—H1*WA*⋯O5^ii^	0.89	2.44	3.320 (17)	169
O1*WA*—H2*WA*⋯O1*WA* ^v^	0.91	2.02	2.92 (3)	169
O1*WB*—H2*WB*⋯O7^ii^	0.88	1.56	2.441 (19)	175

Symmetry codes: (i) 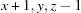; (ii) 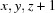; (iii) 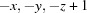; (iv) 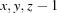; (v) 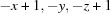.

**Fig. 1.**
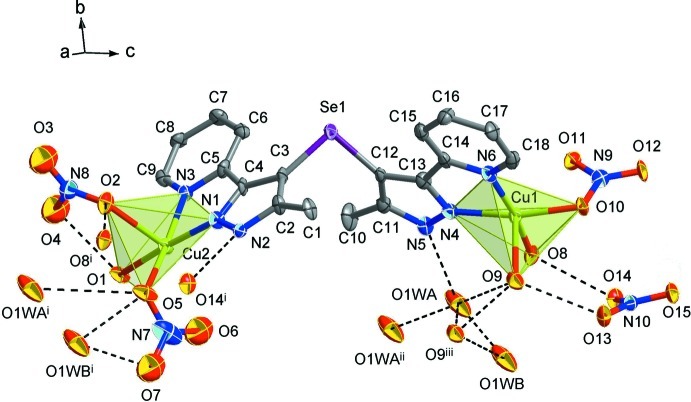
The molecular structure of the title compound, with 50% probability displacement ellipsoids. Hydrogen bonds are indicated by dashed lines. H atoms are omitted for clarity. [Symmetry codes: (i) x, y, -1+z; (ii) 1-x, -y, 1-z; (iii) -x, -y, 1-z.]

**Fig. 2.**
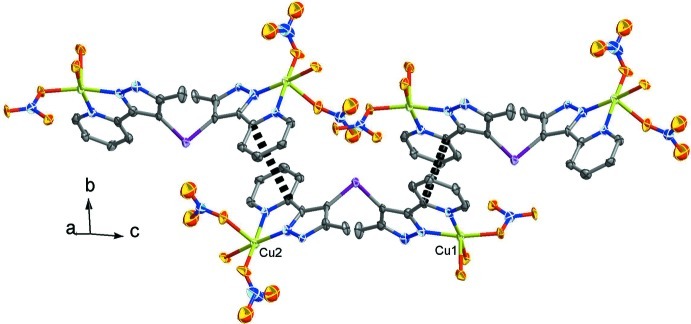
A part of the crystal packing showing π–π stacking interactions between the pyridine rings (dashed lines).

## supplementary crystallographic information

### Comment

Pyrazole-derived ligands are widely used in molecular magnetism, bioinorganic modelling and supramolecular chemistry due to their bridging nature and possibility for easy functionalization (Fritsky *et al.*, 2003; Kovbasyuk *et al.*, 2004; Krämer & Fritsky, 2000; Krämer *et al.*, 2002; Penkova *et al.*, 2009; Sachse *et al.*, 2008). As a part of our synthetic and structural study of pyrazolylselenides (Seredyuk *et al.*, 2010*a*, 2011, 2012) and their complexes with *d*-metals (Seredyuk *et al.*, 2007, 2009, 2010**b*, c*), we report here the crystal structure of the title compound.

The title compound is a binuclear complex (Fig. 1) formed by bis[3-methyl-5-(pyridin-2-yl)-1*H*-pyrazol-4-yl]selenide (Seredyuk *et al.*, 2010*a*). Each Cu^II^ ion is surrounded by three O atoms and two N atoms in a coordination geometry best described as tetragonal pyramidal. For both Cu^II^ ions, the equatorial planes are formed by a chelating pyrazole-pyridine group [Cu—N = 1.961 (7)–2.015 (7) Å], a water molecule [Cu1—O8 = 1.966 (6), Cu2—O1 = 1.978 (6) Å] and a nitrate O atom [Cu1—O10 = 1.988 (6), Cu2—O5 = 1.979 (7) Å], whereas the apical positions are occupied by a water molecule for Cu1 [Cu1—O9 = 2.254 (7) Å] and by a nitrate O atom for Cu2 [Cu2—O2 = 2.345 (7) Å]. The organic selenide adopts a *trans* configuration with a C–Se–C angle equal to 97.9 (4)°. The C—N and C—C bond lengths in the pyridine rings are normal for 2-substituted pyridine derivatives (Fritsky *et al.*, 2004; Kanderal *et al.*, 2005; Moroz *et al.*, 2010, 2012).

An uncoordinated nitrate anion balancing the charge of the complex molecule serves as a bridge to connect three complex molecules through O—H···O and N—H···O hydrogen bonds (Table 1). Also, numerous intermolecular hydrogen bonds are observed between the water molecules and nitrate anions. π–π stacking interactions between the pyridine rings [averaged centroid–centroid distance = 3.652 (5) Å] are also present (Fig. 2).

### Experimental

In a solution of Cu(NO_3_)_2_.6H_2_O (0.144 g, 0.468 mmol) in 5 ml of water a batch of bis[3-methyl-5-(pyridin-2-yl)-1*H*-pyrazol-4-yl]selenide methanol monosolvate (0.1 g, 0.234 mmol) (Seredyuk *et al.*, 2010*a*) was dissolved. After several weeks,well formed blue-green crystals were formed and isolated. Analysis, calculated for C_18_H_26_Cu_2_N_9_O_14_Se: C 27.07, H 3.28, N 15.79%; found: C 27.15, H 3.14, N 15.70%.

### Refinement

H atoms on NH groups and the coordinated water molecules were located from a difference Fourier map and constrained to ride on their parent atoms, with *U*_iso_(H) = 1.2 or 1.5*U*_eq_(N, O). One of the H atoms attached to the coordinated water molecule O9 was found to be disordered over two positions with an occupancy ratio of 0.5:0.5 (as O9 forms a hydrogen bond with its symmetry-related water molecule through H2O9, which limits the occupancy by 1/2). Lattice water molecule was found to be disordered over two sites (O1WA and O1WB), with an occupancy ratio of 0.5:0.5. O1WA and O1WB were restrained with effective standard deviation 0.01 so that its *U*_ij_ components approximate to isotropic behavior. H atoms of the disordered water molecule were placed at calculated positions (Nardelli, 1999) and refined as riding in as-found relative positions with *U*_iso_(H) = 1.5*U*_eq_(O). C-bound H atoms were positioned geometrically and refined as riding atoms, with C—H = 0.93 (CH) and 0.96 (CH_3_) Å and *U*_iso_(H) = 1.2(1.5 for methyl)*U*_eq_(C). Noticeable thermal vibrations of O atoms were observed in some of the nitrate anions, so that geometric constraints were placed on some of the nitrate O atoms to improve their geometries and thermal ellipsoid parameters. The highest residual electron density was found 0.88 Å from O7, and the deepest hole 0.27 Å from O7.

### Crystal data

**Table:**

[Cu_2_(NO_3_)_3_(C_18_H_16_N_6_Se)(H_2_O)_3_]NO_3_·H_2_O	*Z* = 2
*M**_r_* = 842.51	*F*(000) = 844
Triclinic, *P*1	*D*_x_ = 1.889 Mg m^−^^3^
Hall symbol: -P 1	Mo *K*α radiation, λ = 0.71073 Å
*a* = 10.102 (2) Å	Cell parameters from 6135 reflections
*b* = 11.629 (2) Å	θ = 3.5–28.4°
*c* = 12.796 (3) Å	µ = 2.76 mm^−^^1^
α = 98.56 (3)°	*T* = 100 K
β = 93.07 (3)°	Block, green
γ = 93.04 (3)°	0.27 × 0.23 × 0.13 mm
*V* = 1481.5 (5) Å^3^	

### Data collection

**Table:**

Bruker APEXII CCD diffractometer	6511 independent reflections
Radiation source: fine-focus sealed tube	5817 reflections with *I* > 2σ(*I*)
Graphite monochromator	*R*_int_ = 0.073
φ and ω scans	θ_max_ = 28.4°, θ_min_ = 3.5°
Absorption correction: multi-scan (*SADABS*; Sheldrick, 1996)	*h* = −13→13
*T*_min_ = 0.476, *T*_max_ = 0.716	*k* = −15→15
11167 measured reflections	*l* = −17→17

### Refinement

**Table:**

Refinement on *F*^2^	Primary atom site location: structure-invariant direct methods
Least-squares matrix: full	Secondary atom site location: difference Fourier map
*R*[*F*^2^ > 2σ(*F*^2^)] = 0.080	Hydrogen site location: inferred from neighbouring sites
*wR*(*F*^2^) = 0.223	H-atom parameters constrained
*S* = 1.02	*w* = 1/[σ^2^(*F*_o_^2^) + (0.1001*P*)^2^] where *P* = (*F*_o_^2^ + 2*F*_c_^2^)/3
6511 reflections	(Δ/σ)_max_ = 0.002
418 parameters	Δρ_max_ = 2.05 e Å^−^^3^
39 restraints	Δρ_min_ = −1.35 e Å^−^^3^

### Special details

**Table:**

Geometry. All e.s.d.'s (except the e.s.d. in the dihedral angle between two l.s. planes) are estimated using the full covariance matrix. The cell e.s.d.'s are taken into account individually in the estimation of e.s.d.'s in distances, angles and torsion angles; correlations between e.s.d.'s in cell parameters are only used when they are defined by crystal symmetry. An approximate (isotropic) treatment of cell e.s.d.'s is used for estimating e.s.d.'s involving l.s. planes.
Refinement. Refinement of *F*^2^ against ALL reflections. The weighted *R*-factor *wR* and goodness of fit *S* are based on *F*^2^, conventional *R*-factors *R* are based on *F*, with *F* set to zero for negative *F*^2^. The threshold expression of *F*^2^ > σ(*F*^2^) is used only for calculating *R*-factors(gt) *etc*. and is not relevant to the choice of reflections for refinement. *R*-factors based on *F*^2^ are statistically about twice as large as those based on *F*, and *R*- factors based on ALL data will be even larger.

### Fractional atomic coordinates and isotropic or equivalent isotropic displacement parameters (Å^2^)

**Table:**

	*x*	*y*	*z*	*U*_iso_*/*U*_eq_	Occ. (<1)
Se1	0.21129 (9)	0.43349 (8)	0.24378 (7)	0.0235 (3)	
Cu1	0.02084 (10)	0.23134 (10)	0.60026 (9)	0.0206 (3)	
Cu2	0.44191 (10)	0.14805 (10)	−0.10606 (9)	0.0216 (3)	
O1	0.5970 (6)	0.0816 (5)	−0.1753 (5)	0.0272 (16)	
H1O1	0.5992	0.1308	−0.2182	0.041*	
H2O1	0.6854	0.0643	−0.1449	0.041*	
O2	0.3959 (6)	0.2470 (6)	−0.2494 (5)	0.0316 (16)	
O3	0.4542 (10)	0.3529 (9)	−0.3672 (9)	0.0775 (16)	
O4	0.5925 (10)	0.2392 (9)	−0.3114 (9)	0.0775 (16)	
O5	0.3321 (6)	0.0230 (6)	−0.1982 (6)	0.0372 (18)	
O6	0.3899 (10)	−0.0632 (9)	−0.0650 (10)	0.0775 (16)	
O7	0.2943 (10)	−0.1626 (9)	−0.2145 (9)	0.0775 (16)	
O8	0.1528 (6)	0.1420 (6)	0.6666 (5)	0.0258 (15)	
H1O8	0.1165	0.0833	0.6890	0.039*	
H2O8	0.2230	0.1798	0.7126	0.039*	
O9	−0.1007 (6)	0.0624 (6)	0.5480 (5)	0.0289 (15)	
H1O9	−0.1127	0.0330	0.6044	0.043*	
H2O9	−0.0410	0.0253	0.5155	0.043*	0.50
H3O9	−0.1677	0.0810	0.5121	0.043*	0.50
O10	−0.0814 (6)	0.2541 (5)	0.7294 (5)	0.0211 (14)	
O11	0.0904 (6)	0.3763 (5)	0.7686 (5)	0.0254 (15)	
O12	−0.0468 (7)	0.3494 (6)	0.8898 (5)	0.0302 (16)	
O13	−0.1565 (6)	−0.0198 (6)	0.7310 (5)	0.0289 (16)	
O14	0.0347 (6)	0.0339 (6)	0.8173 (5)	0.0245 (15)	
O15	−0.1499 (6)	0.0426 (6)	0.8989 (5)	0.0266 (15)	
O1WA	0.3744 (16)	0.0262 (14)	0.5467 (12)	0.052 (3)	0.50
H1WA	0.3750	0.0219	0.6157	0.078*	0.50
H2WA	0.4560	0.0074	0.5262	0.078*	0.50
O1WB	0.2742 (16)	−0.1094 (14)	0.6081 (12)	0.052 (3)	0.50
H1WB	0.2220	−0.1701	0.5659	0.078*	0.50
H2WB	0.2854	−0.1259	0.6728	0.078*	0.50
N1	0.2979 (7)	0.2094 (6)	−0.0216 (6)	0.0208 (17)	
N2	0.1677 (7)	0.1782 (6)	−0.0076 (6)	0.0191 (16)	
H1N2	0.1358	0.1221	−0.0461	0.023*	
N3	0.5397 (7)	0.2878 (6)	−0.0177 (6)	0.0188 (16)	
N4	0.1334 (7)	0.2424 (7)	0.4797 (6)	0.0222 (17)	
N5	0.2575 (7)	0.2116 (7)	0.4554 (6)	0.0228 (17)	
H1N	0.3032	0.1655	0.4873	0.027*	
N6	−0.0912 (7)	0.3259 (7)	0.5166 (6)	0.0224 (17)	
N7	0.3367 (10)	−0.0720 (9)	−0.1549 (10)	0.062 (3)	
N8	0.4817 (9)	0.2826 (8)	−0.3083 (7)	0.037 (2)	
N9	−0.0101 (7)	0.3290 (7)	0.7998 (6)	0.0243 (18)	
N10	−0.0915 (7)	0.0180 (6)	0.8146 (6)	0.0224 (17)	
C1	−0.0184 (8)	0.2275 (8)	0.1079 (7)	0.021 (2)	
H1A	−0.0662	0.1677	0.0582	0.031*	
H1B	−0.0611	0.2994	0.1085	0.031*	
H1C	−0.0175	0.2055	0.1773	0.031*	
C2	0.1202 (8)	0.2428 (8)	0.0766 (6)	0.0164 (18)	
C3	0.2234 (9)	0.3198 (8)	0.1218 (7)	0.022 (2)	
C4	0.3337 (8)	0.2966 (8)	0.0588 (7)	0.0184 (18)	
C5	0.4711 (8)	0.3463 (8)	0.0583 (7)	0.0198 (19)	
C6	0.5225 (9)	0.4453 (7)	0.1248 (7)	0.0203 (19)	
H6	0.4725	0.4836	0.1766	0.024*	
C7	0.6512 (8)	0.4853 (8)	0.1111 (7)	0.0213 (19)	
H7	0.6889	0.5514	0.1547	0.026*	
C8	0.7243 (9)	0.4275 (8)	0.0331 (7)	0.024 (2)	
H8	0.8102	0.4548	0.0232	0.029*	
C9	0.6673 (8)	0.3289 (7)	−0.0294 (7)	0.0178 (18)	
H9	0.7164	0.2890	−0.0810	0.021*	
C10	0.4320 (9)	0.2365 (10)	0.3298 (9)	0.039 (3)	
H10A	0.4872	0.2059	0.3812	0.059*	
H10B	0.4744	0.3068	0.3132	0.059*	
H10C	0.4185	0.1802	0.2667	0.059*	
C11	0.2990 (9)	0.2627 (8)	0.3746 (7)	0.0216 (19)	
C12	0.1988 (9)	0.3295 (7)	0.3456 (7)	0.0189 (19)	
C13	0.0964 (8)	0.3147 (8)	0.4154 (7)	0.021 (2)	
C14	−0.0378 (8)	0.3594 (8)	0.4312 (7)	0.0181 (18)	
C15	−0.1049 (9)	0.4238 (8)	0.3645 (7)	0.024 (2)	
H15	−0.0666	0.4441	0.3048	0.029*	
C16	−0.2299 (9)	0.4566 (8)	0.3895 (8)	0.024 (2)	
H16	−0.2769	0.5015	0.3478	0.029*	
C17	−0.2853 (9)	0.4213 (8)	0.4786 (8)	0.027 (2)	
H17	−0.3695	0.4428	0.4964	0.032*	
C18	−0.2157 (8)	0.3552 (8)	0.5394 (7)	0.021 (2)	
H18	−0.2542	0.3300	0.5971	0.025*	

### Atomic displacement parameters (Å^2^)

**Table:**

	*U*^11^	*U*^22^	*U*^33^	*U*^12^	*U*^13^	*U*^23^
Se1	0.0247 (5)	0.0220 (5)	0.0236 (5)	−0.0016 (4)	0.0123 (4)	0.0003 (4)
Cu1	0.0187 (6)	0.0248 (6)	0.0192 (6)	0.0027 (5)	0.0057 (4)	0.0040 (5)
Cu2	0.0157 (6)	0.0244 (6)	0.0249 (6)	0.0036 (5)	0.0069 (5)	0.0016 (5)
O1	0.016 (3)	0.030 (4)	0.035 (4)	0.008 (3)	0.009 (3)	−0.002 (3)
O2	0.022 (4)	0.041 (4)	0.034 (4)	0.004 (3)	0.010 (3)	0.009 (3)
O3	0.069 (3)	0.074 (3)	0.101 (4)	0.022 (3)	0.039 (3)	0.032 (3)
O4	0.069 (3)	0.074 (3)	0.101 (4)	0.022 (3)	0.039 (3)	0.032 (3)
O5	0.023 (4)	0.027 (4)	0.059 (5)	0.007 (3)	0.008 (3)	−0.007 (4)
O6	0.069 (3)	0.074 (3)	0.101 (4)	0.022 (3)	0.039 (3)	0.032 (3)
O7	0.069 (3)	0.074 (3)	0.101 (4)	0.022 (3)	0.039 (3)	0.032 (3)
O8	0.021 (3)	0.035 (4)	0.023 (4)	0.004 (3)	0.002 (3)	0.008 (3)
O9	0.034 (4)	0.029 (4)	0.022 (4)	0.004 (3)	−0.001 (3)	−0.003 (3)
O10	0.023 (3)	0.025 (3)	0.014 (3)	−0.002 (3)	0.001 (3)	−0.003 (3)
O11	0.019 (3)	0.028 (4)	0.030 (4)	−0.001 (3)	0.006 (3)	0.005 (3)
O12	0.037 (4)	0.039 (4)	0.015 (3)	0.012 (3)	0.013 (3)	−0.001 (3)
O13	0.029 (4)	0.038 (4)	0.018 (4)	−0.005 (3)	0.006 (3)	0.002 (3)
O14	0.011 (3)	0.035 (4)	0.027 (4)	0.003 (3)	0.005 (3)	0.005 (3)
O15	0.026 (4)	0.036 (4)	0.018 (3)	0.006 (3)	0.009 (3)	0.001 (3)
O1WA	0.063 (6)	0.054 (5)	0.036 (5)	0.032 (5)	−0.013 (4)	−0.008 (4)
O1WB	0.063 (6)	0.054 (5)	0.036 (5)	0.032 (5)	−0.013 (4)	−0.008 (4)
N1	0.007 (3)	0.026 (4)	0.029 (4)	0.002 (3)	0.004 (3)	0.001 (3)
N2	0.017 (4)	0.018 (4)	0.023 (4)	0.000 (3)	0.008 (3)	0.003 (3)
N3	0.012 (4)	0.022 (4)	0.023 (4)	0.003 (3)	0.005 (3)	0.003 (3)
N4	0.016 (4)	0.023 (4)	0.028 (4)	0.004 (3)	0.007 (3)	0.003 (3)
N5	0.014 (4)	0.029 (4)	0.024 (4)	0.000 (3)	0.000 (3)	0.002 (3)
N6	0.014 (4)	0.026 (4)	0.027 (4)	0.005 (3)	0.004 (3)	0.003 (3)
N7	0.036 (5)	0.047 (6)	0.094 (7)	−0.010 (5)	0.026 (5)	−0.022 (6)
N8	0.041 (6)	0.035 (5)	0.038 (5)	0.007 (4)	0.013 (4)	0.011 (4)
N9	0.015 (4)	0.027 (4)	0.032 (5)	0.004 (3)	0.002 (3)	0.005 (4)
N10	0.021 (4)	0.020 (4)	0.028 (5)	0.002 (3)	0.010 (3)	0.006 (3)
C1	0.007 (4)	0.036 (5)	0.019 (5)	0.009 (4)	0.006 (3)	−0.001 (4)
C2	0.006 (4)	0.030 (5)	0.013 (4)	0.002 (3)	−0.001 (3)	0.000 (4)
C3	0.023 (5)	0.027 (5)	0.016 (5)	0.000 (4)	0.013 (4)	0.002 (4)
C4	0.016 (4)	0.026 (5)	0.014 (4)	0.004 (4)	0.010 (3)	0.004 (3)
C5	0.012 (4)	0.029 (5)	0.021 (5)	0.000 (4)	0.006 (4)	0.008 (4)
C6	0.021 (5)	0.019 (5)	0.021 (5)	0.008 (4)	0.003 (4)	0.003 (4)
C7	0.013 (4)	0.021 (5)	0.027 (5)	−0.003 (4)	0.000 (4)	−0.002 (4)
C8	0.019 (5)	0.023 (5)	0.032 (6)	−0.004 (4)	0.009 (4)	0.005 (4)
C9	0.012 (4)	0.022 (5)	0.019 (5)	0.006 (3)	0.002 (3)	0.001 (4)
C10	0.016 (5)	0.065 (8)	0.039 (7)	0.009 (5)	0.016 (5)	0.011 (6)
C11	0.019 (5)	0.023 (5)	0.022 (5)	−0.001 (4)	0.004 (4)	0.000 (4)
C12	0.022 (5)	0.018 (5)	0.016 (4)	−0.005 (4)	0.010 (4)	−0.002 (3)
C13	0.012 (4)	0.022 (5)	0.026 (5)	−0.004 (4)	0.005 (4)	0.000 (4)
C14	0.017 (4)	0.022 (5)	0.013 (4)	−0.003 (4)	0.004 (3)	−0.005 (3)
C15	0.023 (5)	0.027 (5)	0.022 (5)	0.001 (4)	0.009 (4)	−0.002 (4)
C16	0.019 (5)	0.027 (5)	0.030 (5)	0.007 (4)	0.007 (4)	0.014 (4)
C17	0.021 (5)	0.032 (6)	0.028 (5)	0.007 (4)	0.014 (4)	0.003 (4)
C18	0.016 (4)	0.026 (5)	0.019 (5)	−0.007 (4)	0.003 (4)	0.002 (4)

### Geometric parameters (Å, º)

**Table:**

Se1—C3	1.903 (9)	N3—C5	1.348 (10)
Se1—C12	1.909 (9)	N3—C9	1.374 (10)
Cu1—O8	1.966 (6)	N4—C13	1.315 (11)
Cu1—N4	1.980 (7)	N4—N5	1.361 (10)
Cu1—O10	1.988 (6)	N5—C11	1.342 (11)
Cu1—N6	1.998 (7)	N5—H1N	0.8600
Cu1—O9	2.254 (7)	N6—C14	1.342 (11)
Cu2—N1	1.961 (7)	N6—C18	1.356 (10)
Cu2—O1	1.978 (6)	C1—C2	1.486 (11)
Cu2—O5	1.979 (7)	C1—H1A	0.9600
Cu2—N3	2.015 (7)	C1—H1B	0.9600
Cu2—O2	2.345 (7)	C1—H1C	0.9600
O1—H1O1	0.8502	C2—C3	1.380 (12)
O1—H2O1	0.9965	C3—C4	1.425 (11)
O2—N8	1.270 (10)	C4—C5	1.476 (11)
O3—N8	1.225 (12)	C5—C6	1.384 (12)
O4—N8	1.252 (12)	C6—C7	1.386 (12)
O5—N7	1.309 (12)	C6—H6	0.9300
O6—N7	1.231 (12)	C7—C8	1.389 (12)
O7—N7	1.247 (12)	C7—H7	0.9300
O8—H1O8	0.8521	C8—C9	1.376 (12)
O8—H2O8	0.9420	C8—H8	0.9300
O9—H1O9	0.8544	C9—H9	0.9300
O9—H2O9	0.8510	C10—C11	1.516 (12)
O9—H3O9	0.8534	C10—H10A	0.9600
O10—N9	1.311 (9)	C10—H10B	0.9600
O11—N9	1.242 (9)	C10—H10C	0.9600
O12—N9	1.222 (9)	C11—C12	1.377 (12)
O13—N10	1.233 (10)	C12—C13	1.423 (11)
O14—N10	1.276 (9)	C13—C14	1.490 (11)
O15—N10	1.261 (9)	C14—C15	1.390 (13)
O1WA—H1WA	0.8913	C15—C16	1.379 (12)
O1WA—H2WA	0.9065	C15—H15	0.9300
O1WB—H1WB	0.9407	C16—C17	1.402 (12)
O1WB—H2WB	0.8807	C16—H16	0.9300
N1—C4	1.352 (11)	C17—C18	1.366 (13)
N1—N2	1.373 (9)	C17—H17	0.9300
N2—C2	1.344 (10)	C18—H18	0.9300
N2—H1N2	0.7989		
			
C3—Se1—C12	97.9 (4)	O11—N9—O10	116.5 (7)
O8—Cu1—N4	91.0 (3)	O13—N10—O15	119.9 (7)
O8—Cu1—O10	92.5 (3)	O13—N10—O14	121.1 (7)
N4—Cu1—O10	168.5 (3)	O15—N10—O14	119.0 (8)
O8—Cu1—N6	170.8 (3)	C2—C1—H1A	109.5
N4—Cu1—N6	79.9 (3)	C2—C1—H1B	109.5
O10—Cu1—N6	96.1 (3)	H1A—C1—H1B	109.5
O8—Cu1—O9	88.0 (3)	C2—C1—H1C	109.5
N4—Cu1—O9	102.8 (3)	H1A—C1—H1C	109.5
O10—Cu1—O9	88.3 (2)	H1B—C1—H1C	109.5
N6—Cu1—O9	95.3 (3)	N2—C2—C3	106.4 (7)
N1—Cu2—O1	173.1 (3)	N2—C2—C1	123.3 (8)
N1—Cu2—O5	96.5 (3)	C3—C2—C1	130.3 (8)
O1—Cu2—O5	86.7 (3)	C2—C3—C4	106.5 (8)
N1—Cu2—N3	80.1 (3)	C2—C3—Se1	124.5 (6)
O1—Cu2—N3	97.3 (3)	C4—C3—Se1	129.1 (7)
O5—Cu2—N3	173.7 (3)	N1—C4—C3	109.3 (8)
N1—Cu2—O2	96.1 (3)	N1—C4—C5	114.7 (7)
O1—Cu2—O2	90.4 (3)	C3—C4—C5	136.0 (8)
O5—Cu2—O2	81.3 (3)	N3—C5—C6	123.4 (8)
N3—Cu2—O2	93.7 (3)	N3—C5—C4	112.4 (8)
Cu2—O1—H1O1	93.2	C6—C5—C4	124.1 (8)
Cu2—O1—H2O1	131.0	C5—C6—C7	117.5 (8)
H1O1—O1—H2O1	115.1	C5—C6—H6	121.3
N8—O2—Cu2	125.3 (6)	C7—C6—H6	121.3
N7—O5—Cu2	108.0 (7)	C6—C7—C8	120.6 (8)
Cu1—O8—H1O8	111.7	C6—C7—H7	119.7
Cu1—O8—H2O8	121.2	C8—C7—H7	119.7
H1O8—O8—H2O8	113.1	C9—C8—C7	118.8 (8)
Cu1—O9—H1O9	105.7	C9—C8—H8	120.6
Cu1—O9—H2O9	97.5	C7—C8—H8	120.6
H1O9—O9—H2O9	107.5	N3—C9—C8	121.8 (8)
Cu1—O9—H3O9	104.7	N3—C9—H9	119.1
H1O9—O9—H3O9	119.4	C8—C9—H9	119.1
H2O9—O9—H3O9	118.8	C11—C10—H10A	109.5
N9—O10—Cu1	106.7 (5)	C11—C10—H10B	109.5
H1WA—O1WA—H2WA	106.2	H10A—C10—H10B	109.5
H1WB—O1WB—H2WB	109.3	C11—C10—H10C	109.5
C4—N1—N2	105.2 (7)	H10A—C10—H10C	109.5
C4—N1—Cu2	115.9 (5)	H10B—C10—H10C	109.5
N2—N1—Cu2	137.9 (6)	N5—C11—C12	107.0 (7)
C2—N2—N1	112.7 (7)	N5—C11—C10	120.4 (8)
C2—N2—H1N2	130.9	C12—C11—C10	132.5 (9)
N1—N2—H1N2	116.0	C11—C12—C13	105.7 (8)
C5—N3—C9	118.0 (7)	C11—C12—Se1	124.9 (6)
C5—N3—Cu2	115.9 (6)	C13—C12—Se1	129.0 (7)
C9—N3—Cu2	126.1 (6)	N4—C13—C12	109.1 (8)
C13—N4—N5	107.3 (7)	N4—C13—C14	115.0 (7)
C13—N4—Cu1	115.8 (6)	C12—C13—C14	135.9 (9)
N5—N4—Cu1	135.4 (6)	N6—C14—C15	122.6 (8)
C11—N5—N4	110.9 (7)	N6—C14—C13	112.0 (8)
C11—N5—H1N	124.5	C15—C14—C13	125.3 (8)
N4—N5—H1N	124.6	C16—C15—C14	118.0 (8)
C14—N6—C18	119.4 (8)	C16—C15—H15	121.0
C14—N6—Cu1	116.2 (6)	C14—C15—H15	121.0
C18—N6—Cu1	124.4 (6)	C15—C16—C17	119.1 (9)
O6—N7—O7	127.9 (13)	C15—C16—H16	120.4
O6—N7—O5	117.4 (10)	C17—C16—H16	120.4
O7—N7—O5	114.4 (12)	C18—C17—C16	120.1 (8)
O3—N8—O4	119.6 (10)	C18—C17—H17	120.0
O3—N8—O2	120.7 (9)	C16—C17—H17	120.0
O4—N8—O2	119.5 (9)	N6—C18—C17	120.8 (8)
O12—N9—O11	124.5 (8)	N6—C18—H18	119.6
O12—N9—O10	119.0 (7)	C17—C18—H18	119.6
			
N1—Cu2—O2—N8	148.5 (7)	N2—N1—C4—C3	−0.7 (9)
O1—Cu2—O2—N8	−29.2 (7)	Cu2—N1—C4—C3	−171.5 (6)
O5—Cu2—O2—N8	−115.8 (7)	N2—N1—C4—C5	−178.7 (7)
N3—Cu2—O2—N8	68.1 (7)	Cu2—N1—C4—C5	10.6 (10)
N1—Cu2—O5—N7	−92.2 (6)	C2—C3—C4—N1	0.3 (10)
O1—Cu2—O5—N7	81.7 (6)	Se1—C3—C4—N1	−179.0 (7)
O2—Cu2—O5—N7	172.6 (6)	C2—C3—C4—C5	177.7 (10)
O8—Cu1—O10—N9	72.0 (5)	Se1—C3—C4—C5	−1.7 (16)
N4—Cu1—O10—N9	−35.9 (16)	C9—N3—C5—C6	0.1 (13)
N6—Cu1—O10—N9	−105.0 (5)	Cu2—N3—C5—C6	−178.8 (7)
O9—Cu1—O10—N9	159.9 (5)	C9—N3—C5—C4	177.1 (7)
O5—Cu2—N1—C4	176.4 (6)	Cu2—N3—C5—C4	−1.9 (9)
N3—Cu2—N1—C4	−9.0 (6)	N1—C4—C5—N3	−5.5 (11)
O2—Cu2—N1—C4	−101.7 (6)	C3—C4—C5—N3	177.2 (10)
O5—Cu2—N1—N2	9.8 (9)	N1—C4—C5—C6	171.4 (8)
N3—Cu2—N1—N2	−175.5 (9)	C3—C4—C5—C6	−5.8 (16)
O2—Cu2—N1—N2	91.7 (9)	N3—C5—C6—C7	0.1 (13)
C4—N1—N2—C2	0.9 (9)	C4—C5—C6—C7	−176.5 (8)
Cu2—N1—N2—C2	168.4 (7)	C5—C6—C7—C8	0.3 (13)
N1—Cu2—N3—C5	5.8 (6)	C6—C7—C8—C9	−0.9 (14)
O1—Cu2—N3—C5	−167.8 (6)	C5—N3—C9—C8	−0.8 (12)
O2—Cu2—N3—C5	101.3 (6)	Cu2—N3—C9—C8	178.0 (7)
N1—Cu2—N3—C9	−173.1 (7)	C7—C8—C9—N3	1.2 (13)
O1—Cu2—N3—C9	13.4 (7)	N4—N5—C11—C12	0.3 (10)
O2—Cu2—N3—C9	−77.5 (7)	N4—N5—C11—C10	−176.9 (8)
O8—Cu1—N4—C13	−169.3 (7)	N5—C11—C12—C13	0.4 (10)
O10—Cu1—N4—C13	−61.3 (17)	C10—C11—C12—C13	177.2 (10)
N6—Cu1—N4—C13	9.3 (6)	N5—C11—C12—Se1	173.5 (6)
O9—Cu1—N4—C13	102.5 (7)	C10—C11—C12—Se1	−9.8 (15)
O8—Cu1—N4—N5	−5.6 (8)	C3—Se1—C12—C11	67.1 (8)
O10—Cu1—N4—N5	102.4 (15)	C3—Se1—C12—C13	−121.5 (8)
N6—Cu1—N4—N5	173.0 (9)	N5—N4—C13—C12	1.2 (10)
O9—Cu1—N4—N5	−93.7 (8)	Cu1—N4—C13—C12	169.3 (6)
C13—N4—N5—C11	−1.0 (10)	N5—N4—C13—C14	−179.3 (7)
Cu1—N4—N5—C11	−165.6 (7)	Cu1—N4—C13—C14	−11.2 (10)
N4—Cu1—N6—C14	−5.6 (6)	C11—C12—C13—N4	−1.0 (10)
O10—Cu1—N6—C14	163.4 (6)	Se1—C12—C13—N4	−173.7 (7)
O9—Cu1—N6—C14	−107.7 (6)	C11—C12—C13—C14	179.6 (10)
N4—Cu1—N6—C18	172.9 (8)	Se1—C12—C13—C14	6.9 (15)
O10—Cu1—N6—C18	−18.0 (7)	C18—N6—C14—C15	0.2 (13)
O9—Cu1—N6—C18	70.8 (7)	Cu1—N6—C14—C15	178.9 (7)
Cu2—O5—N7—O6	8.7 (12)	C18—N6—C14—C13	−177.2 (7)
Cu2—O5—N7—O7	−166.3 (8)	Cu1—N6—C14—C13	1.5 (9)
Cu2—O2—N8—O3	−165.3 (8)	N4—C13—C14—N6	6.3 (11)
Cu2—O2—N8—O4	19.6 (13)	C12—C13—C14—N6	−174.4 (9)
Cu1—O10—N9—O12	−173.5 (6)	N4—C13—C14—C15	−171.0 (9)
Cu1—O10—N9—O11	7.8 (8)	C12—C13—C14—C15	8.4 (16)
N1—N2—C2—C3	−0.7 (10)	N6—C14—C15—C16	1.7 (14)
N1—N2—C2—C1	179.2 (8)	C13—C14—C15—C16	178.7 (8)
N2—C2—C3—C4	0.2 (10)	C14—C15—C16—C17	−1.7 (14)
C1—C2—C3—C4	−179.6 (9)	C15—C16—C17—C18	−0.1 (14)
N2—C2—C3—Se1	179.6 (6)	C14—N6—C18—C17	−2.1 (13)
C1—C2—C3—Se1	−0.3 (14)	Cu1—N6—C18—C17	179.4 (7)
C12—Se1—C3—C2	70.2 (8)	C16—C17—C18—N6	2.0 (14)
C12—Se1—C3—C4	−110.6 (9)		

### Hydrogen-bond geometry (Å, º)

**Table:**

*D*—H···*A*	*D*—H	H···*A*	*D*···*A*	*D*—H···*A*
O1—H1*O*1···O4	0.85	1.86	2.710 (12)	176
O1—H2*O*1···O15^i^	1.00	1.77	2.763 (9)	174
O8—H1*O*8···O14	0.85	2.02	2.744 (9)	142
O8—H2*O*8···O2^ii^	0.94	1.88	2.772 (9)	156
O9—H1*O*9···O13	0.85	1.88	2.732 (9)	173
O9—H2*O*9···O9^iii^	0.85	1.94	2.794 (9)	175
O9—H3*O*9···O1*WA*^iii^	0.85	2.40	3.024 (18)	131
O9—H3*O*9···O1*WB*^iii^	0.85	1.91	2.723 (16)	159
N2—H1*N*2···O14^iv^	0.80	2.08	2.822 (10)	155
N5—H1*N*···O1*WA*	0.86	2.04	2.878 (18)	166
O1*WA*—H1*WA*···O5^ii^	0.89	2.44	3.320 (17)	169
O1*WA*—H2*WA*···O1*WA*^v^	0.91	2.02	2.92 (3)	169
O1*WB*—H2*WB*···O7^ii^	0.88	1.56	2.441 (19)	175

Symmetry codes: (i) *x*+1, *y*, *z*−1; (ii) *x*, *y*, *z*+1; (iii) −*x*, −*y*, −*z*+1; (iv) *x*, *y*, *z*−1; (v) −*x*+1, −*y*, −*z*+1.
